# *Vibrio cholerae* O1 Variant with Reduced Susceptibility to Ciprofloxacin, Western Africa

**DOI:** 10.3201/eid1611.100568

**Published:** 2010-11

**Authors:** Marie-Laure Quilici, Denis Massenet, Bouba Gake, Barem Bwalki, David M. Olson

**Affiliations:** Author affiliations: Institut Pasteur, Paris, France (M.-L. Quilici);; Centre Pasteur Cameroun, Garoua, Cameroon (D. Massenet, B. Gake);; State Epidemiologic Unit, Yola, Adamawa State, Nigeria (B. Bwalki);; Doctors Without Borders/Médecins Sans Frontières, New York, New York, USA (D. M. Olson)

**Keywords:** Cholera, Vibrio cholerae, bacteria, antimicrobial drug susceptibility, ciprofloxacin, Africa, Nigeria, Cameroon, letter

**To the Editor:** Many variants of choleragenic vibrios have emerged since the beginning of the seventh pandemic, indicating continuous evolution of this pathogenic agent. Variations occur mainly in genetic determinants of virulence and antimicrobial drug susceptibility. In September–October 2009, concurrent outbreaks of acute watery diarrhea in northeastern Nigeria (4,559 cases) and northern Cameroon (696 cases) were investigated by state ministries of health. We report reduced sensitivity to ciprofloxacin in *Vibrio cholerae* O1 strains and the atypical cholera toxin B (*ctxB*) genotype of these strains.

In September–October 2009, stool specimens from patients in Nigeria were collected on filter paper, moistened with sterile physiologic saline, and sent at room temperature to the National Reference Center for Vibrios and Cholera at the Institut Pasteur (Paris, France). Ten *V. cholerae* O1 biotype El Tor serotype Ogawa strains were isolated and identified by using standard procedures. Concurrently in Cameroon, 9 *V. cholerae* O1 Ogawa strains isolated from patient stool samples by the bacteriology laboratory of the Pasteur Center (Garoua, Cameroon) were sent to the National Reference Center for Vibrios and Cholera.

All strains were tested for antimicrobial susceptibility by MIC determination to tetracycline, trimethoprim/sulfamethoxazole, sulfonamides, ampicillin, chloramphenicol, nalidixic acid, and ciprofloxacin by using Etest (AB bioMérieux, Solna, Sweden) according to Clinical and Laboratory Standards Institute procedures and interpretative standards for *V. cholerae* (*1*). PCR amplification of the genes encoding DNA gyrase (*gyrA* and *gyrB*) and topoisomerase IV (*parC* and *parE*) and subsequent sequencing of PCR products were performed (*2*).

PCR was used to test for the presence of *ctxA* and *ctxB* genes, which encode the cholera toxin (CT), and the *tcpA* gene, which encodes the toxin-coregulated pilus. Genotyping of *ctxB* was performed by sequencing PCR products.

All isolates showed susceptibility to tetracycline (MIC 1.5 mg/L), intermediate susceptibility to ampicillin (MICs 12–16 mg/L) and chloramphenicol (MICs 8–12 mg/L), and resistance to trimethoprim/sulfamethoxazole (MIC >32 mg/L), sulfonamides (MIC >1,024 mg/L), and nalidixic acid (MIC >256 mg/L). MICs of ciprofloxacin ranged from 0.25 to 0.5 mg/L.

Sequencing of *gyrA*, *gyrB*, *parC*, and *parE* genes among all strains detected 1 mutation in *gyrA* (substitution of serine by isoleucine at position 83) and 1 mutation in *parC* (substitution of serine by leucine at position 85). Both point mutations have been associated with quinolone resistance in clinical isolates of *V. cholerae* (*2*). None of the strains had any mutations in *gyrB* or *parE*.

The presence of *ctxA* and *ctxB* genes confirmed the toxigenicity of all isolates, and *tcpA* PCR product size and sequence identified El Tor biotype strains. The DNA sequence of *ctxB* was similar to that of the recently reported Orissa variant identified in India in 2007 (*3*). This sequence had 2 mutations resulting in histidine at position 39 and threonine at position 68 (this amino acid sequence is similar to the CT-B subunit of the reference classical strain) and a third mutation resulting in substitution of histidine by asparagine at position 20.

We report atypical El Tor strains of *V. cholerae* O1 and their reduced susceptibility to ciprofloxacin in Nigeria and Cameroon. Since the 1990s, atypical El Tor strains that produce classical CT have been increasingly reported from countries in Asia, where they have gradually replaced the prototype El Tor strains, but they have only been reported in 2 countries in Africa (Mozambique and Zambia) (*4**,**5*). On the basis of the CT-B subunit sequence, these variants differ from variants isolated in southern Africa and from most variants isolated in Asia by having the same modified classical CT as a strain recently isolated in Orissa in eastern India (*3*), which has not been reported elsewhere. These findings indicate evolution of *V. cholerae* O1 El Tor hybrid strains. Their presence may indicate spread of strains from eastern India to Africa (*6*).

The presence of CT-B variants in central or western Africa is of great concern because these strains may be more toxigenic (*3*). There is also concern for the strains isolated in this study because of their reduced susceptibility to ciprofloxacin. Although reduced susceptibility to fluoroquinolone is common in southern Asia (*7**,**8*), it was reported in Africa (Zimbabwe) only recently (*9*). Our findings, in addition to the report of Islam et al. (*9*), indicate that *V*. *cholera* with reduced sensitivity to a fluoroquinolone is present in southern and western Africa. These results highlight the need for continued monitoring of antimicrobial drug susceptibility and strain tracking to maintain an efficient cholera surveillance system.
